# Exposure to restrictive diet content on social media and dietary behaviors among women aged 18–49 years in Poland

**DOI:** 10.3389/fpubh.2026.1941731

**Published:** 2026-09-18

**Authors:** Mateusz Grajek, Patrycja Pers, Tomasz Jurys, Mateusz Rozmiarek

**Affiliations:** 1 Department of Public Health, Faculty of Public Health in Bytom, Medical University of Silesia in Katowice, Bytom, Poland; 2 Department of Rehabilitation, Faculty of Health Sciences in Katowice, Medical University of Silesia in Katowice, Katowice, Poland; 3 Department of Sports Tourism, Faculty of Physical Culture Sciences, Poznan University of Physical Education, Poznan, Poland

**Keywords:** body image, dietary behavior, digital health literacy, eating behavior, restrictive diets, social media, TFEQ-13, women

## Abstract

**Background:**

Social media have become one of the primary sources of nutrition-related information among young adults; however, the widespread promotion of restrictive diets may contribute to unhealthy eating behaviors and body image concerns. The aim of this study was to examine the association between exposure to restrictive diet-related content on social media and eating-behavior dimensions measured using the TFEQ-13 among women aged 18–49 years in Poland. Additionally, the study descriptively assessed participants’ restrictive dietary practices and their self-reported perceptions regarding the influence of social media on dietary decisions, self-esteem, and body image.

**Methods:**

A cross-sectional survey was conducted among 370 women using an original questionnaire and the Three-Factor Eating Questionnaire (TFEQ-13). The questionnaire assessed exposure to diet-related content, restrictive dietary practices, sources of nutrition information, body image, and eating-related behaviors.

**Results:**

Most respondents reported exposure to diet-related social media content several times daily (70.8%), with Instagram and TikTok representing the primary information sources. Nearly half of the participants had followed a diet promoted on social media, most commonly involving sugar elimination or intermittent fasting. Rapid weight loss was the leading motivation for adopting restrictive diets. Although physicians and dietitians were perceived as the most trusted sources of nutrition information, participants frequently reported perceiving social media content as relevant to their dietary decisions, body image, and eating-related attitudes. Self-reported body dissatisfaction, guilt after eating foods perceived as unhealthy, and restrictive or compensatory eating practices were common in the sample. In inferential analyses, greater exposure to restrictive diet-related social media content was positively associated with cognitive restraint and uncontrolled eating after adjustment for age, educational level, place of residence, and previous experience with restrictive diets promoted on social media, whereas the adjusted association with emotional eating was not statistically significant.

**Conclusion:**

Frequent exposure to restrictive diet-related social media content was associated with higher cognitive restraint and, to a lesser extent, uncontrolled eating. Body dissatisfaction, restrictive dietary practices, and potentially maladaptive eating-related attitudes were also frequently reported but were assessed descriptively and should not be interpreted as outcomes statistically associated with social media exposure. Given the cross-sectional design, the observed associations do not establish causality.

## Introduction

Dietary behaviors represent one of the key determinants of health, influencing both the prevention and development of chronic diseases, as well as overall physical and psychological wellbeing. Diet is commonly defined as an individual pattern of food consumption that considers both the quantity and quality of consumed products (1). Its primary purpose is to provide the organism with essential nutrients while ensuring that their intake is adapted to physiological requirements, including digestion, absorption, and metabolism (2). Therefore, an appropriate dietary pattern should not only meet nutritional requirements but also support long-term health maintenance and disease prevention.

Contemporary approaches to nutrition emphasize that dietary patterns should be considered within a broader interaction between diet, health, and environmental sustainability. This perspective contributed to the development of the concept of healthy sustainable diets (HSD), introduced by the Food and Agriculture Organization (FAO), which describes dietary patterns that ensure nutritional adequacy while minimizing environmental impact and supporting food security for current and future generations (3, 4). The concept of healthy sustainable diets integrates nutritional adequacy, health promotion, environmental sustainability, and socio-cultural acceptability, emphasizing the need to consider dietary patterns beyond individual nutritional outcomes (5). A balanced diet should provide adequate amounts of macronutrients, including carbohydrates, proteins, and fatty acids, as well as vitamins, minerals, and bioactive compounds necessary for proper functioning of the body (4). However, in addition to health-promoting dietary patterns, modern societies are increasingly exposed to various alternative dietary approaches, including restrictive diets, which are frequently promoted as rapid solutions for weight reduction or achieving an ideal body shape.

Restrictive diets are dietary patterns characterized by significant limitations in energy intake or the exclusion of specific food groups or nutrients. Restrictive eating behaviors may also involve avoidance of specific foods or food groups, which in some individuals may be related to food neophobia or avoidant/restrictive eating patterns (6). Although some forms of dietary restriction may be clinically justified when implemented under professional supervision, such as therapeutic diets adapted to specific medical conditions, self-imposed restrictive eating patterns may pose significant health risks (4, 7). Popular restrictive diets promoted outside medical indications often include very low-calorie diets, single-food diets, low-carbohydrate high-fat diets, and highly restrictive vegetarian or vegan patterns (4). Their increasing popularity is particularly concerning because many of these approaches are disseminated through non-scientific sources, where potential benefits are frequently emphasized while possible adverse effects are minimized.

Long-term adherence to restrictive dietary patterns may lead to insufficient intake of essential nutrients, nutritional deficiencies, impaired physiological functioning, and deterioration of overall health (2). Moreover, restrictive eating behaviors may be associated with excessive preoccupation with food quality, health-oriented eating concerns, and altered motivations related to dietary control and physical activity (8). Such behaviors may overlap with orthorexic tendencies, characterized by an excessive focus on healthy eating and rigid dietary rules, although orthorexia nervosa remains a debated diagnostic construct (9, 10). Furthermore, repeated engagement in restrictive dieting has been identified as a potential risk factor for the development of disordered eating patterns, particularly among adolescents and young adults (11). The risk may be particularly relevant among women aged 18–49 years, a population frequently exposed to social pressure regarding appearance, body weight, and dietary control. Although appropriately planned vegetarian and vegan diets may provide health benefits and contribute to the prevention of some chronic diseases, poorly balanced versions may increase the risk of deficiencies in nutrients such as vitamin B12, iron, calcium, and omega-3 fatty acids (4, 8). Previous research indicates that inadequate restrictive diets may negatively affect cognitive functioning, psychological health, and quality of life, particularly when adopted without professional nutritional guidance (12, 13). The relationship between nutrition and mental health is bidirectional, as dietary patterns may influence mood, cognitive processes, and psychological wellbeing, while mental health conditions may also affect eating behaviors and food choices (14).

In recent years, social media platforms have become one of the most influential sources of information regarding nutrition, health, and lifestyle behaviors. Digital environments enable rapid dissemination of dietary advice, personal experiences, and visual representations of body ideals, often reaching large populations of adolescents and young adults. The growing influence of social media on health-related behaviors has become an important public health issue, particularly because online nutritional content is frequently created by individuals without formal expertise in nutrition or medicine (15, 16). Unlike traditional sources of health information, social media allows users to interact directly with content creators, including influencers, whose recommendations may shape followers’ attitudes, beliefs, and behaviors.

The relationship between media exposure and dietary behaviors is complex. On one hand, digital platforms may promote positive health behaviors, such as increased awareness of balanced nutrition, physical activity, and disease prevention. On the other hand, social media may contribute to the normalization of restrictive eating practices and unrealistic expectations regarding body appearance (17). Studies indicate that media frequently present conflicting messages by simultaneously promoting highly processed foods and idealized standards of thinness (17). Such contradictory communication may increase confusion regarding appropriate dietary choices and encourage individuals to seek extreme methods of weight control.

Among women aged 18–49 years, the influence of social media may be particularly significant due to the strong association between appearance, self-worth, and social acceptance. Contemporary media frequently promote a culturally dominant ideal of a slim, attractive, and highly controlled body, which may contribute to dissatisfaction with one’s own appearance and increased engagement in weight-control behaviors (17). Exposure to idealized images and body-related content has been associated with negative body image, reduced self-esteem, and increased risk of disturbed eating behaviors (18, 19). Recent evidence from a scoping review indicates that social media use may contribute to body image concerns and eating disorder symptomatology among young people, particularly through mechanisms such as social comparison, internalization of appearance ideals, and exposure to appearance-focused content (20). Moreover, recent findings further indicate that digital media use may play an important role in shaping body esteem and self-perception among adolescents, with gender-related differences influencing the magnitude of these associations (21). In addition, online communities promoting extreme approaches to weight loss, including pro-anorexia (pro-ana) movements, demonstrate how digital spaces can reinforce harmful beliefs about food restriction and body control, particularly among women aged 18–49 years (17, 22).

Despite the increasing availability of nutrition-related information on social media, the extent to which restrictive diet content influences women aged 18–49 years’s dietary behaviors, self-perception, and psychological wellbeing remains unclear. Given the potential impact of digital environments on health-related decision-making, investigating the relationship between exposure to restrictive dietary messages and eating-related outcomes represents an important public health issue. Therefore, further research is needed to better understand how social media content, particularly messages promoted by influencers, may contribute to both adaptive and maladaptive dietary behaviors.

The primary aim of the present study was to examine whether the frequency of exposure to restrictive diet-related content on social media was associated with the three dimensions of eating behavior measured by the Three-Factor Eating Questionnaire (TFEQ-13) among women aged 18–49 years in Poland. A secondary, descriptive aim was to characterize participants’ restrictive dietary practices and their self-reported perceptions of the influence of social media on dietary decisions, self-esteem, and body image. These questionnaire-based perceptions were treated as descriptive outcomes and were not included as dependent variables in the inferential association analyses.

## Materials and methods

### Study design and setting

A cross-sectional study was conducted between January and May 2026 using an anonymous self-administered questionnaire. The study was designed primarily to examine associations between exposure to restrictive diet-related social media content and eating-behavior dimensions assessed using the TFEQ-13. The original questionnaire additionally provided descriptive information on restrictive dietary practices and participants’ self-reported perceptions concerning dietary decisions, self-esteem, and body image. The study procedures were non-experimental and did not involve any medical intervention.

### Participants and recruitment

Participants were recruited using a non-probability purposive sampling strategy between January and May 2026. The survey was distributed electronically via the Google Forms platform through selected social media channels, including thematic groups and profiles focused on nutrition, health, physical activity, and lifestyle. To increase the diversity of the study population, the questionnaire was also disseminated through academic communities and student networks across Poland. In addition, a snowball sampling approach was applied, whereby participants were encouraged to share the survey link with other women who met the eligibility criteria. Participation was entirely voluntary, anonymous, and uncompensated.

Women aged 18–49 years were eligible for inclusion in the study. This age range was selected to capture adult women of reproductive age (23, 24), a population group frequently examined in research on body image, dieting, eating behaviors, and social media use. The inclusion criteria comprised female sex, age between 18 and 49 years, the ability to understand the questionnaire, and the provision of informed consent. Exclusion criteria included age below 18 or above 49 years, incomplete questionnaire responses, duplicate submissions, and failure to meet the predefined eligibility criteria.

A total of 400 questionnaire submissions were received during the study period. Because recruitment was conducted through open online dissemination, academic networks, social media channels, and snowball sampling, the total number of individuals who viewed or received the survey invitation could not be determined. Therefore, a conventional survey response rate could not be calculated. Of the 400 submitted questionnaires, 370 (92.5%) met the eligibility and data-quality criteria and were included in the final analysis. Thirty submissions were excluded: 18 because of incomplete questionnaire data, 8 because the respondent did not meet the predefined eligibility criteria, and 4 because they were identified as duplicate submissions. Potential duplicate submissions were identified by comparing automatically generated response identifiers, submission timestamps, and the pattern of responses across key demographic and questionnaire variables; records showing identical or near-identical response patterns submitted within a short time interval were manually reviewed and classified as duplicates when sufficient concordance was observed.

### Research instrument

Data were collected using an original questionnaire developed for the purpose of this study and the standardized Three-Factor Eating Questionnaire (TFEQ-13).

The questionnaire consisted of three sections: (1) Sociodemographic characteristics—including questions regarding basic participant characteristics; (2) Original questionnaire regarding social media use and dietary behaviors, including single-choice and multiple-choice questions, as well as items based on a five-point Likert scale. The scale ranged from 1 (“strongly disagree”/“minimal influence”) to 5 (“strongly agree”/“maximum influence”). The questionnaire assessed, among others, time spent on social media platforms, types of nutrition- and diet-related content followed online, exposure to restrictive diet-related messages, and perceived influence of social media content on self-esteem and dietary behaviors; (3) Three-Factor Eating Questionnaire (TFEQ-13)—a shortened validated version of the original TFEQ, consisting of 13 statements assessing eating-related behaviors, including cognitive restraint, uncontrolled eating, and emotional eating.

The TFEQ-13 was scored according to the scoring procedure described for the Polish adaptation of the instrument (25). Items were assigned to three subscales: cognitive restraint, uncontrolled eating, and emotional eating. Negatively keyed items were reverse-coded where applicable. For each participant, each subscale score was calculated as the mean of the items assigned to that subscale, with higher scores indicating a greater intensity of the corresponding eating-behavior dimension. The theoretical score range was 1.00–4.00 for each subscale. In the present sample, the observed score ranges were 1.00–4.00 for cognitive restraint, 1.00–3.80 for uncontrolled eating, and 1.00–4.00 for emotional eating. The mean scores were 2.76 (SD = 0.64) for cognitive restraint, 2.18 (SD = 0.57) for uncontrolled eating, and 1.93 (SD = 0.71) for emotional eating. No diagnostic cut-off points were applied because the TFEQ-13 assesses dimensions of eating behavior rather than providing a diagnosis of an eating disorder. Internal consistency in the present sample was acceptable for cognitive restraint (*ω* = 0.81, *α* = 0.79), acceptable for uncontrolled eating (*ω* = 0.83, *α* = 0.81), and moderate for emotional eating (*ω* = 0.71, *α* = 0.69). Corrected item–total correlations ranged from 0.31 to 0.68. Although the emotional eating subscale demonstrated slightly lower internal consistency, the reliability coefficients were considered acceptable for research purposes given the limited number of items.

### Data collection procedure and statistical analysis

Data were screened for completeness and consistency before analysis. Descriptive statistics were calculated for all study variables. Categorical variables were presented as frequencies and percentages, whereas continuous variables were summarized using means, standard deviations (SD), medians, interquartile ranges (IQR), and observed ranges, as appropriate. The Three-Factor Eating Questionnaire (TFEQ-13) was scored according to the procedure described for the Polish adaptation of the instrument. Items were assigned to the cognitive restraint, uncontrolled eating, and emotional eating subscales. Negatively keyed items were reverse-coded prior to calculating the subscale scores. Internal consistency was assessed separately for each subscale using McDonald’s omega (*ω*) and Cronbach’s alpha (*α*), together with corrected item–total correlations. Categorical variables were summarized using frequencies and percentages. No inferential tests were applied solely to determine whether nominal response categories differed from an arbitrary equal expected distribution. Associations between the frequency of exposure to restrictive diet-related content on social media and the TFEQ-13 subscale scores were initially examined using Spearman’s rank correlation coefficients (*ρ*). Correlation coefficients were reported together with 95% confidence intervals. To evaluate the independent association between social media exposure and eating behavior dimensions, separate multivariable linear regression models were constructed for cognitive restraint, uncontrolled eating, and emotional eating. The primary explanatory variable was the reported frequency of exposure to restrictive diet-related content on social media. The models were adjusted for age, educational level, place of residence, and previous experience with restrictive diets promoted on social media. Regression results are presented as unstandardized regression coefficients (*B*), standardized coefficients (*β*), 95% confidence intervals (95% CI), and *p*-values. Adjusted coefficients of determination (adjusted *R*^2^) were calculated to estimate the proportion of explained variance. All correlation and multivariable regression analyses reported in Tables 1 and 2 were performed using the correctly calculated TFEQ-13 mean-item subscale scores described above. Statistical significance was set at *p* < 0.05. All statistical analyses were performed using Statistica version 13.3 (TIBCO Software Inc., Palo Alto, CA, USA).

**Table 1:** Associations between exposure to restrictive diet-related content on social media and TFEQ-13 subscales.

TFEQ-13 subscale	Spearman’s *ρ*	95% CI	*p*-value
Cognitive restraint	0.34	0.24–0.43	<0.001
Uncontrolled eating	0.21	0.11–0.31	<0.001
Emotional eating	0.18	0.08–0.28	0.001

**Table 2:** Multivariable linear regression models adjusted for age, educational level, place of residence, and previous experience with restrictive diets promoted on social media.

Outcome variable	*B*	95% CI for *B*	Standardized *β*	*p*-value	Adjusted *R*^2^
Cognitive restraint	0.16	0.10–0.22	0.31	<0.001	0.14
Uncontrolled eating	0.08	0.03–0.13	0.16	0.004	0.08
Emotional eating	0.04	−0.01–0.09	0.08	0.150	0.05

### Ethical considerations

The study was conducted in accordance with the ethical principles outlined in the Declaration of Helsinki and complied with all applicable national regulations governing research involving human participants. Participation was voluntary and anonymous. Before accessing the questionnaire, participants were presented with an online information sheet describing the purpose of the study, the voluntary nature of participation, anonymity and confidentiality, and their right to discontinue participation before submitting the questionnaire. Consent was implied by the participant’s voluntary continuation to and submission of the anonymous online questionnaire after reading the study information. No personally identifiable information was collected.

The study was conducted as a continuation of a broader research program approved by the Bioethics Committee of the Medical University of Silesia in Katowice in 2022 (approval No. PCN/CBN/0052/KB/127/22). The original approval covered research conducted using psychometric scales and questionnaire-based methods relevant to the assessment of eating behaviors. The present study, including the questionnaire-based assessment and the use of the TFEQ-13, was carried out within the scope of this approved research framework.

## Results

The study included 370 women aged 18–49 years. Nearly half of the respondents lived in cities with more than 100,000 inhabitants (49.2%), while 31.4% resided in towns with 10,000–100,000 inhabitants and 19.5% in rural areas. Most participants had secondary education (61.6%), followed by higher education (32.4%), whereas vocational (5.4%) and primary education (0.5%) were reported only occasionally. These findings indicate that the study population consisted predominantly of urban women with at least secondary education (Table 3).

**Table 3:** Characteristics of the study participants.

Characteristic	*n* (%)/descriptive statistic
Age, years	
Mean ± SD	29.8 ± 8.1
Median (IQR)	28.0 (23.0–35.0)
Range	18–49
Age group	
18–24 years	119 (32.2)
25–34 years	137 (37.0)
35–44 years	82 (22.2)
45–49 years	32 (8.6)
Place of residence	
Rural areas	72 (19.5)
Towns (10,000–100,000 inhabitants)	116 (31.4)
Cities (>100,000 inhabitants)	182 (49.2)
Educational level	
Primary/vocational	22 (5.9)
Secondary	228 (61.6)
Higher	120 (32.4)

Regarding exposure to nutrition-related information, the vast majority of respondents reported encountering social media content concerning diets, weight loss, or healthy lifestyles several times a day (70.8%). Considerably fewer participants declared exposure several times a week (17.0%), once a day (8.7%), or once a week (3.5%). Instagram (40.0%) and TikTok (35.7%) constituted the primary sources of dietary information, whereas YouTube (10.0%), Facebook (9.5%), Pinterest (3.8%), and PubMed (0.8%) were selected substantially less frequently—Table 4.

**Table 4:** Social media exposure and restrictive dieting behaviors.

Variable	Category	*n* (%)
Frequency of exposure to diet-related content	Several times a day	262 (70.8)
	Several times a week	63 (17.0)
	Once a day	32 (8.7)
	Once a week	13 (3.5)
Following diets promoted on social media	Yes	177 (47.8)
	No	193 (52.2)
Type of diet followed^*^	Sugar and sweets elimination	66 (37.3)
	Intermittent fasting	52 (29.4)
	Ketogenic diet	17 (9.6)
	Juice detox	16 (9.0)
	Gluten-free diet without medical indication	11 (6.2)
	Vegetarian or vegan diet	10 (5.7)
	Other	5 (2.8)
Duration of diet^*^	Less than 1 week	25 (14.1)
	1–4 weeks	80 (45.2)
	More than 1 month	72 (40.7)
Motivation for restrictive dieting^*^	Rapid weight loss	95 (53.7)
	Perceived health improvement based on social media claims	46 (26.0)
	Appearance-related pressure	29 (16.4)
	Following influencer-promoted trends	7 (4.0)
Purchasing influencer-promoted products	Yes	109 (29.5)
	No	261 (70.5)
Type of purchased product^*^	Dietary supplements	65 (59.6)
	Workout plans	17 (15.6)
	Diet catering services	15 (13.8)
	Cookbooks or recipe e-books	10 (9.2)
	Dietary plans	2 (1.8)

^*^Variables coded so that higher values indicate greater exposure or stronger endorsement.

Analysis of the types of content viewed on social media demonstrated that recipes for “healthy” meals were the most frequently followed (42.4%), followed by motivational messages related to exercise and body shape (20.5%) and before-and-after body transformation posts (17.8%). Advice regarding supplementation and detoxification (11.9%) and promotion of specific diets (7.3%) were considerably less common. Nearly half of the respondents (47.8%) admitted having tried a diet based on information obtained from social media. Among women who had followed such diets, eliminating sugar and sweets (37.3%) and intermittent fasting (29.4%) were the most frequently reported dietary approaches, whereas ketogenic diets (9.6%), juice detoxes (9.0%), gluten-free diets without medical indications (6.2%), vegetarian or vegan diets (5.7%), and other diets (2.8%) were substantially less common.

Among participants who had followed diets promoted through social media, the most frequently reported duration was between one and 4 weeks (45.2%), followed by more than 1 month (40.7%), whereas only 14.1% reported following such diets for less than 1 week. The leading motivation for adopting these diets was rapid weight loss (53.7%), followed by improving health according to social media claims (26.0%). Appearance-related pressure (16.4%) and following influencer-promoted trends (4.0%) were reported considerably less often. Nearly one-third of respondents (29.4%) declared purchasing products recommended by influencers or other social media users, whereas 70.6% had never done so. Among purchased products, dietary supplements predominated (59.6%), followed by workout plans (15.4%), diet catering services (13.5%), cookbooks or recipe e-books (9.6%), and dietary plans (1.9%). Analysis of self-reported dietary practices showed that respondents most frequently excluded fats (37.3%) from their diet, followed by carbohydrates (33.5%) and proteins (25.4%).

When evaluating factors influencing dietary decisions, respondents reported that advice provided by physicians and dietitians exerted the strongest influence, with approximately 80% selecting the categories “rather influences” or “definitely influences.” Social media represented the second most influential source, whereas traditional media were perceived as having the weakest impact—Table 5.

**Table 5:** Perceived influence of social media on eating attitudes.

Variable	Mean ± SD	Median (IQR)
Physician/dietitian influences dietary decisions	4.15 ± 1.12	5 (4–5)
Social media influence dietary decisions	3.15 ± 1.43	4 (2–4)
Social media negatively affect self-esteem	3.41 ± 1.51	4 (2–4)
Feeling guilty after eating unhealthy food	2.86 ± 1.63	3 (1–4)
Restrictive diets promote healthy eating	1.68 ± 1.03	1 (1–2)
Social media increase restrictive eating	3.04 ± 1.47	4 (2–4)

Respondents demonstrated relatively critical attitudes toward restrictive dietary behaviors promoted online. Most participants disagreed that restrictive diets contribute to maintaining a healthy relationship with food, whereas many agreed that frequent exposure to diet-related content negatively affected their self-esteem and body image. Furthermore, a considerable proportion acknowledged feelings of guilt after eating foods perceived as unhealthy and recognized that browsing social media increased their willingness to adopt more restrictive eating habits.

Behavioral indicators of restrictive eating demonstrated that calorie or macronutrient counting represented the most common compensatory behavior, whereas avoiding social gatherings because of dietary concerns occurred least frequently. The need to compensate for consuming “unhealthy” foods through exercise or dietary restriction was also relatively common. Evaluation of body image indicated generally low satisfaction among participants. More than half of respondents reported being unhappy or very unhappy with both their body shape and current body weight, whereas only a small minority declared being very satisfied.

The mean TFEQ-13 score was highest for cognitive restraint (*M* = 2.76, SD = 0.64), followed by uncontrolled eating (*M* = 2.18, SD = 0.57) and emotional eating (*M* = 1.93, SD = 0.71). Median scores were 2.75 (IQR: 2.25–3.25) for cognitive restraint, 2.20 (IQR: 1.80–2.60) for uncontrolled eating, and 2.00 (IQR: 1.33–2.33) for emotional eating. All subscales were expressed as mean item scores on the same theoretical scale ranging from 1.00 to 4.00. Detailed descriptive statistics are presented in Table 6 and 7.

**Table 6:** Eating behaviors and body image outcomes.

Variable	Mean ± SD	Median (IQR)
Calorie/macronutrient counting	2.62 ± 1.62	2 (1–4)
Need to “burn off” calories	2.85 ± 1.39	3 (2–4)
Avoiding social eating	1.85 ± 1.28	1 (1–2)
Compensatory behaviors	2.06 ± 1.35	1 (1–3)
Body shape satisfaction	2.90 ± 1.05	3 (2–4)
Body weight satisfaction	2.74 ± 1.18	2 (2–4)

**Table 7:** Descriptive statistics for TFEQ-13 subscale scores.

TFEQ-13 subscale	Mean ± SD	Median (IQR)	Theoretical range	Observed range
Cognitive restraint	2.76 ± 0.64	2.75 (2.25–3.25)	1.00–4.00	1.00–4.00
Uncontrolled eating	2.18 ± 0.57	2.20 (1.80–2.60)	1.00–4.00	1.00–3.80
Emotional eating	1.93 ± 0.71	2.00 (1.33–2.33)	1.00–4.00	1.00–4.00

Spearman’s rank correlation analyses indicated that more frequent exposure to restrictive diet-related content on social media was positively associated with all three TFEQ-13 dimensions. The strongest association was observed for cognitive restraint (*ρ* = 0.34, 95% CI: 0.24–0.43, *p* < 0.001). Weaker positive associations were observed for uncontrolled eating (*ρ* = 0.21, 95% CI: 0.11–0.31, *p* < 0.001) and emotional eating (*ρ* = 0.18, 95% CI: 0.08–0.28, *p* = 0.001)—Table 1.

In multivariable regression analyses adjusted for age, educational level, place of residence, and previous experience with restrictive diets promoted on social media, greater exposure to restrictive diet-related content remained significantly associated with higher cognitive restraint (*B* = 0.16, 95% CI: 0.10–0.22, standardized *β* = 0.31, *p* < 0.001). A weaker independent association was observed for uncontrolled eating (*B* = 0.08, 95% CI: 0.03–0.13, standardized *β* = 0.16, *p* = 0.004). In contrast, the adjusted association between exposure frequency and emotional eating was not statistically significant (*B* = 0.04, 95% CI: −0.01 to 0.09, standardized *β* = 0.08, *p* = 0.150). The models explained 14, 8, and 5% of the variance in cognitive restraint, uncontrolled eating, and emotional eating, respectively—Table 2.

## Discussion

The results of the present study revealed a notable discrepancy between participants’ declared trust in different sources of nutrition information and their reported dietary behaviors. Respondents reported the highest levels of trust in physicians and dietitians, while information shared on social media was generally evaluated as less reliable. At the same time, participants reported behaviors such as self-directed elimination of macronutrients, purchasing dietary supplements based on influencer recommendations, and experiencing an internal need to “burn off” consumed meals. These findings indicate that social media constitutes an important context in which women aged 18–49 years encounter and potentially engage with diet-related information; however, the present cross-sectional data do not allow us to determine whether social media exposure directly caused these behaviors.

The additional analyses demonstrated that women reporting more frequent exposure to restrictive diet-related content on social media also tended to report higher levels of cognitive restraint and, to a lesser extent, uncontrolled eating. After adjustment for selected sociodemographic characteristics, only the association with cognitive restraint remained relatively strong, suggesting that exposure to restrictive dietary content may be related primarily to greater intentional restriction of food intake rather than emotional eating. Nevertheless, because of the cross-sectional design, these findings should be interpreted as statistical associations rather than evidence of causality.

One possible interpretation, consistent with previous literature, is that the perceived relatability and accessibility of influencers may increase the salience of their recommendations, even when users simultaneously report greater trust in professional health sources (26). Thus, the discrepancy observed in the present study may reflect a difference between general trust in professional sources and the practical accessibility or attractiveness of information encountered through social media. Previous research has suggested that influencer-generated content, including simplified dietary messages, personal success stories, and appearance-oriented material, may shape attitudes and intentions related to eating and weight management, particularly among young adults (27).

A key descriptive aspect of this study concerned dietary behaviors and self-reported body image perceptions among women aged 18–49 years in the context of frequent exposure to diet-related social media content. Body image outcomes were not included in the inferential association analyses and should therefore be interpreted descriptively. These findings can be considered in the context of previous research examining diet-related social media use. Turner and Lefevre, in a study of 680 social media users who followed diet-related accounts, reported an association between the frequency of social media use and the severity of orthorexia symptoms (28). In the present study, 71% of respondents reported encountering diet-related content several times a day, and a substantial proportion reported negative effects of such content on self-esteem and body image. Although these observations are broadly consistent with previous research linking frequent exposure to diet-related social media content with eating- and body-image-related concerns, the present study did not assess orthorexia symptoms directly and therefore cannot establish that the reported behaviors represent the same phenomenon. Similarly, the present analyses did not examine whether exposure to influencer-generated content preceded or caused restrictive or compensatory behaviors. Nevertheless, the coexistence of frequent exposure to diet-related content and reported restrictive or compensatory practices warrants attention and is consistent with the broader literature suggesting that social media may provide an environment in which rigid dietary norms and appearance-related expectations are encountered frequently (28, 29). Previous research has proposed that the visual and appearance-oriented nature of social media may contribute to social comparison and body dissatisfaction (29). This provides one possible theoretical framework for interpreting the present findings, but this mechanism was not directly examined in the current study. Accordingly, the reported associations should be understood as being compatible with, rather than demonstrating, such a mechanism. The self-directed elimination of macronutrients and reported compensatory behaviors observed in the present sample may therefore be viewed as behaviors occurring in a social media environment in which appearance- and diet-related content is frequently encountered, rather than as outcomes that can be causally attributed to social media exposure.

More recent evidence further supports the relevance of examining social media use in relation to eating-related outcomes among women aged 18–49 years, while also emphasizing the need for cautious interpretation. In a large cross-sectional study of 4,692 women aged 18–34 years, Altan et al., found significant associations between social media use, eating-related behaviors, BMI, and depressive symptoms. Their analyses indicated that social media use patterns and dietary variables, including snack consumption, were among the variables associated with scores reflecting the influence of social media on eating behavior (30). Importantly, the authors did not interpret these associations as evidence of a causal effect, and emphasized that the observed relationships may reflect differences in platform use, user characteristics, and behavioral patterns (30). These findings provide contemporary support for the relevance of social media as a context associated with eating-related outcomes in women aged 18–49 years and are broadly consistent with the present observation that diet-related social media exposure is common in this population. However, the present study extends this literature by focusing on specific self-reported dietary practices and perceptions of social media content, rather than attempting to establish a causal pathway between social media exposure and eating behavior.

A recent cross-sectional study by Kocaman et al., conducted among 456 women aged 19–32 years, similarly highlights the complexity of the relationship between social media use and eating-related outcomes. The authors found that associations with eating attitudes differed across social media platforms, while correlations between social media use measures and EAT-26 scores were generally weak (31). Notably, neither the duration nor frequency of social media use was significantly associated with EAT-26 scores in their study (31). These findings are relevant to the interpretation of the present results because they suggest that social media exposure should not be treated as a uniform or inherently harmful phenomenon. Instead, the type of content encountered, the platform used, the purpose of engagement, and individual characteristics may all contribute to differences in eating-related outcomes. In the present study, the high frequency of exposure to diet-related content and the reported dietary and body-image concerns should therefore not be interpreted as evidence that the duration of social media use itself caused these behaviors. Rather, the findings support the need to consider the content and context of social media exposure when examining its potential relationship with eating-related behaviors (31).

The potential relationship between the online environment, body image, and eating-related concerns has also been examined by Szaniawska et al. Their findings provide a broader theoretical context for understanding how repeated exposure to idealized appearance standards may be associated with body dissatisfaction and eating-related concerns (32). In the present study, a substantial proportion of respondents reported frequent exposure to diet-related content, as well as feelings of guilt and an internal need to “compensate for” meals perceived as unhealthy. These observations are compatible with previous research linking appearance-related social media exposure with body dissatisfaction and compensatory eating- or exercise-related behaviors. However, the present study did not directly test whether exposure to idealized online content preceded these experiences, nor did it assess clinical eating disorders. Therefore, these findings should be interpreted as indicators of potentially problematic eating-related attitudes and behaviors rather than evidence of an established eating disorder or a causal effect of social media exposure (32, 33).

The perspective proposed by Turner and Lefevre, as well as by Szaniawska and colleagues, is further informed by the findings of Sanzari et al., who emphasized that the type of social media content may be more relevant to body image and disordered eating outcomes than the duration of exposure alone (34). This perspective is relevant to the present study because participants were not only social media users but were frequently exposed to diet-related content. The present findings therefore suggest that the content encountered online may be an important factor to consider when interpreting participants’ dietary and body-image-related responses. However, because the present study did not experimentally distinguish between different types of social media content or assess exposure longitudinally, the results do not demonstrate that particular content caused the reported behaviors. Rather, they are consistent with the broader argument that the quality and nature of online content may be more informative than overall screen time when examining potential relationships between social media and eating-related outcomes (34).

From a public health perspective, the findings highlight the potential importance of nutritional and digital health literacy among young adults. Access to large amounts of health-related information does not necessarily translate into evidence-based dietary behaviors, particularly when users have difficulty evaluating the credibility, commercial background, and scientific basis of online content. Strengthening media literacy may therefore represent a potentially useful preventive approach, although the effectiveness of such interventions should be evaluated directly in future research (35, 36). McLean et al. suggested that critical media literacy may help young people develop a more reflective approach to appearance-related and health-related media content (36). In this context, improving users’ ability to recognize selective presentation, commercial persuasion, and idealized representations may be relevant to reducing susceptibility to misleading dietary messages. However, whether such educational approaches can directly reduce restrictive or compensatory eating behaviors requires further empirical evaluation.

Taken together, the present findings should be interpreted primarily as evidence of the coexistence of frequent exposure to diet-related social media content and a range of reported dietary and body-image-related concerns among women aged 18–49 years. The findings do not establish that social media exposure caused these behaviors, nor do they allow the identification of specific psychological mechanisms underlying the observed associations. Theoretical explanations involving social comparison, influencer credibility, internalization of appearance ideals, or compensatory coping are supported by previous literature and may provide plausible frameworks for interpreting the findings, but these mechanisms were not directly tested in the present study. Future longitudinal and experimental studies should therefore examine whether specific types of social media content precede changes in dietary behaviors, body image, and eating-related attitudes, and should consider potential differences between platforms and patterns of engagement.

### Strenghts and limitations

The present study has several strengths. It addresses a timely and increasingly relevant public health issue by examining the relationship between exposure to restrictive diet-related content on social media and eating-related behaviors among women aged 18–49 years. The study combined a standardized instrument (TFEQ-13) with an original questionnaire assessing social media exposure, dietary practices, and self-reported body image perceptions. The TFEQ-13 enabled inferential assessment of associations between social media exposure and specific eating-behavior dimensions, whereas the remaining questionnaire-based outcomes provided descriptive characterization of the surveyed sample. The psychometric properties of the TFEQ-13 were confirmed in the present sample through internal consistency analyses, supporting the reliability of the obtained subscale scores. Furthermore, the application of multivariable statistical analyses enabled the evaluation of associations between social media exposure and individual dimensions of eating behavior while accounting for selected sociodemographic characteristics.

Nevertheless, several limitations should be acknowledged. First, the cross-sectional design precludes conclusions regarding the temporal direction or causality of the observed associations. Second, all variables were based on self-reported data, which may have been affected by recall bias, reporting bias, and social desirability bias. Third, participants were recruited using a non-probability online sampling strategy through social media, academic networks, and snowball sampling, increasing the risk of selection bias and limiting the representativeness of the study population. Consequently, the findings should not be generalized to all women in Poland. Fourth, although the regression models were adjusted for selected sociodemographic variables, residual confounding cannot be excluded because potentially relevant factors, such as BMI, objectively measured dietary intake, physical activity, previous eating disorder diagnosis, mental health status, and total time spent on social media, were not included in the analyses. Finally, the study included only women aged 18–49 years; therefore, the findings cannot be directly extrapolated to men, adolescents, or older adults.

## Conclusion

Frequent exposure to restrictive diet-related content on social media was independently associated with higher cognitive restraint and, to a lesser extent, uncontrolled eating among women aged 18–49 years. No statistically significant independent association with emotional eating was observed after adjustment for the included covariates. Participants also frequently reported body dissatisfaction, restrictive dietary practices, and potentially maladaptive eating-related attitudes; however, these variables were assessed descriptively and were not formally analyzed as outcomes associated with social media exposure. Accordingly, they should be interpreted as characteristics of the surveyed sample rather than as demonstrated consequences of social media exposure. Given the cross-sectional design and non-probability sampling strategy, the findings do not establish causality and should not be generalized to all women in Poland. Future longitudinal studies are needed to clarify the temporal direction and underlying mechanisms of the observed associations.

## Data Availability

The raw data supporting the conclusions of this article will be made available by the authors, without undue reservation.
